# Quality of Life and Clinical Correlates in Adults with Social Phobia: A Scoping Review

**DOI:** 10.2174/1745017902117010224

**Published:** 2021-12-22

**Authors:** Hui Miin Lau, Kai Samuel Sim, Qian Hui Chew, Kang Sim

**Affiliations:** 1 Monash School of Medicine, Monash University, Melbourne, Australia; 2 Department of Research , Institute of Mental Health, Buangkok Green Medical Park, 539747, Republic of Singapore, Singapore; 3 West Region, Institute of Mental Health, Buangkok Green Medical Park, 539747, Republic of Singapore, Singapore

**Keywords:** Anxiety disorders, Comorbidity, Quality of life, Social phobia, Social anxiety disorder, Correlates

## Abstract

**Objective:**

In light of the substantial clinical and societal burden of social phobia (SP) and impact on the sense of well-being of affected individuals, we sought to summarise extant data related to quality of life and relevant correlates in adults with SP to distill clinical profiles for earlier identification and appropriate management.

**Methods:**

A scoping review was carried out on studies examining quality of life in adults with SP and clinical correlates within different settings. PubMed/Medline and Web of Science databases were searched for relevant articles beginning from database inception until May 2021.

**Results:**

A total of 25 papers were included. Most of the studies (92%) were cross sectional in nature (80%), conducted in the West (92%), and within clinic or community settings (88%). Patients with comorbid psychiatric conditions, and undergraduate students reported higher rates of SP compared with community population. Significant correlates of SP included demographic (such as females, younger age, living alone, fewer years of education, unemployment) and clinical factors (such as family history of anxiety disorders, suicidal ideas, avoidant personality features). SP was widely associated with decreased QoL involving several domains and especially related to complexity, greater number of feared or trigger situations, and comorbid medical and psychiatric conditions.

**Conclusion:**

SP is not uncommon within clinical, and undergraduate populations, and has a significantly negative impact on quality of life. Awareness of its associated clinical profiles allows better identification and overall management of this condition including improvement in QoL.

## 1 INTRODUCTION

Social Phobia (SP), also known as Social Anxiety Disorder, is a potentially debilitating condition characterised by the marked fear or anxiety regarding social or performance situations related to potential scrutiny and negative evaluation by others, with lifetime prevalence estimates of up to 16% in high income countries [1, 2], and can result in functional impairments involving occupational, social and academic domains within affected individuals [1, 3]. In addition, the presence of medical and psychiatric comorbidities may further compound the negative impact of SP on an individual [4-6]. Of note, SP remains under-treated and under-recognised despite its substantial clinical and societal burden, and it was reported that the annual per capita total costs for SP were substantially higher compared with those without mental disorder [7-9]. The nature of the condition also means that individuals suffering from SP are more likely to worry about the social consequences that arise from help-seeking, such as treatment stigma or fear of being negatively judged by healthcare providers [10]. This delay in help-seeking could result in a prolonged and significant impact on their quality of life.

For patients with SP, the quality of life (QoL) can be affected to varying degrees and relates to the personal (self-realisation, work, education, *etc*.) and interpersonal (social relationships) arenas [6]. Over time, there has been increasing interest in examining the health-related QoL amongst individuals with SP within different contexts (such as students, within the community, those with existing medical conditions) [3, 8, 11-13] and related to different subtypes of the condition (such as complexity of fears) [14]. Given that individuals with SP often delay seeking help, an understanding of the interrelationships between QoL and specific demographic and clinical features of SP identified through the review could give clinicians a better idea of what to look out for during routine patient examinations. This provides an opportunity for early identification and intervention so as to optimize the overall clinical management of the disorder. However, to the best of our knowledge, there has been no recent review done that broadly summarizes findings across different contexts and subtypes of SP.

In view of the clinical import of SP and impact on one’s sense of well-being in various domains, we conducted a scoping review to summarise the actual data pertaining to QoL in adults with SP and determine the salient associated clinical features.

## 2 MATERIALS AND METHODS

This scoping review was conducted in accordance with the methodology of the Joanna Briggs Institute for scoping reviews and we followed the five steps framework to guide the process [15, 16]. The first step involves identifying the main research questions addressed within our review, which were:

(1) What are the relevant demographic and clinical correlates of SP?

(2) What is the impact of SP on QoL and associated factors within the sufferers?

The second step involves identifying relevant studies. We searched the PubMed/Medline and Web of Science databases for relevant studies that examined QoL in adults with SP from database inception until May 2021. Keywords and combinations used for the literature search were (quality of life) OR (QOL) AND (adults) AND (social phobia) OR (social anxiety disorder), as well as MeSH (Phobia, Social). Papers were selected for inclusion if they 1) included individuals with SP either within cross-sectional or prospective studies, 2) age group 18 years and older, 3) were focused on examining QoL and 4) were written in English. Papers were excluded if they 1) did not include adults with SP in the sample, and 2) were case reports or opinion pieces.

The third step involves study selection. We manually screened the abstracts of identified reports to ascertain whether they met the inclusion criteria, then reviewed full reports of promising studies. Two independent reviewers simultaneously screened the titles and abstracts. In case of any inconsistency between reviewers, the disagreement was resolved by a third reviewer.

The fourth and fifth steps involve charting, collating, summarizing, and reporting the results. For each included study, we extracted variables including the characteristics of subjects, measure of QOL, and the salient findings. The preceding data was organized within digitalized spreadsheets and then summarized into a table to help facilitate critical assessments and for independent consideration by readers. The preferred reporting items for systematic reviews (PRISMA) flowchart for this review is shown in Fig. (**1**) [17].

## 3 RESULTS

### 3.1 General features of studies and prevalence of SP

We evaluated 40 studies for eligibility and excluded 15 articles due to reasons such as a) child or adolescent sample only, b) QOL was not the main outcome of interest, c) participants with social phobia were a minority in the sample of interest (≤ 1%). Overall, 25 papers were included in this review and the characteristics of these studies are summarised in Table **1**. The majority of the studies were conducted either in the Americas or Europe (N=23, 92%) with a preponderance of cross-sectional studies (N=20, 80%). The nature of the study setting included clinics (N=14, 56%%), community (N=8, 32%) and university undergraduates (N=3, 12%). In terms of prevalence of SP, the rates varied depending on the context. Within community settings, the rates varied from 2.1% to 7.1% [4, 12-14]. Within patients with comorbid conditions such as post-traumatic stress disorder (PTSD) and schizophrenia, the rates ranged between 12.7% to 17% [4, 18]. Within university undergraduates, studies have reported rates of up to 37.6% [3, 8].

### 3.2 Demographic and Clinical Correlates

Regarding gender, SP seems to affect females more than males within the community [4, 13, 14] and undergraduates [3, 8, 19]. In community-based studies, there is greater preponderance of females being associated with SP up to 1.6 fold [14]. Within undergraduates, using logistic regression analysis, one study showed that being female was associated with SP [3], while another showed that the risk of SP was 1.7-fold higher among females than males [19]. In a clinic sample of patients undergoing bariatric surgery with SP, Mirijello *et al.* [20] found that males have a lower propensity than females to meet the cut-off for a diagnosis of SP when measured with the Liebowitz Social Anxiety Scale (LSAS). Pertaining to age, there is some suggestion of SP being associated with younger age in two community based studies [13, 14].

Clinical factors associated with SP included family history of anxiety or psychiatric disorders, history of suicidal ideas/attempts, avoidant personality features [3, 8, 18, 19]. In terms of limited treatment studies, three separate studies which employed Cognitive Behavioural Therapy (CBT) in adults with SP [21, 22] and unguided Internet based self-help intervention [23] amongst undergraduate students with SP respectively observed improvements in QoL upon follow up over time.

Some sociodemographic factors associated with SP included being single/divorced/separated [24], living alone [13] or in cities [19], being less well-educated [13], current tobacco use [3], and embarrassment with own socioeconomic status [8], but not social class or personal income [14].

### 3.3 Impact of Social Situation on QoL

The presence of SP was correlated with an overall decrease in QoL in the majority of included studies [3, 8, 19, 25] and affected specific QoL domains as measured using rating tools. For example, using the Short Form 36 (SF-36), individuals with SP showed greater impairments in the following dimensions, namely, vitality, role limitations related to physical, emotional and social functioning, and mental health [6, 11, 26]. Studies that utilized scales other than SF-36 have also reported problems in these specific QOL domains [12, 25, 27].

Within SP, there exists two different subtypes, namely Generalised Social Phobia (GSP) and Non-Generalised Social Phobia (NGSP) (or Specific SP) [28]. GSP is characterised by the individual with SP facing distress over a wide range of social situations, while NGSP is limited to a few situations (such as public speaking). Comparing across the two subtypes of SP, GSP reported worse QoL relative to NGSP, while NGSP reported QoL that was not significantly different from the healthy control group [28]. In another study, patients who have complex fears subtype of SP (equivalent to GSP) reported poorer QoL compared to the specific public speaking subtype (equivalent to NGSP) [14]. Of note, poorer QoL was associated with a greater number of feared situations in SP, increased comorbidity and service utilization [13]. In addition, the number of trigger situations related to SP was found to be the strongest predictor of reductions in QoL as measured by SF-36, but not the duration of illness, age of onset of SP or depressive scores [26].

### 3.4 Influence of Comorbidities on QOL in the Presence of SP

In terms of comorbidities, patients with medical and psychiatric conditions and co-occurring SP generally suffered from poor QoL [4, 5, 13, 14, 18, 28, 29]. Regarding medical comorbidities, SP was found to be significantly more prevalent and associated with lower QoL scores in patients with vitiligo and acne [29] and morbid obesity awaiting bariatric surgery [20] compared to healthy controls. For example, the percentage of patients showing SP in morbidly obese patients awaiting bariatric surgery was significantly higher (43%) compared to a sample of healthy controls (16%) [20]. Patients awaiting bariatric surgery showed lower QoL scores compared with those who had undergone surgery [20]. In a recent study of patients with hemifacial spasms, those with comorbid SP had lower QoL in domains including vitality, role limitations pertaining to physical and emotional areas [11]. However, 6 months after microvascular decompression for the hemifacial spasm, there were improvements in the anxiety level as well as QoL, which were sustained at 36 months.

Pertaining to co-occurring psychiatric conditions, the prevalence of SP amongst patients with schizophrenia was 17% in an earlier study [18] but lower at 12.7% within patients with PTSD in a more recent study [4]. The comorbidity with mood and anxiety disorders was linearly correlated with the number of social fears, with 92% of respondents with five or six fears reporting a comorbid anxiety disorder, and 56% reporting a comorbid mood disorder [13]. Patients with schizophrenia and SP had lower QoL and associated earlier onset of illness, greater severity of illness, increased depressive symptoms, self-stigmatisation, harm avoidance and lower levels of hope, self-esteem and self-directedness [5, 18]. Of note, lower QOL had a greater association with rates of anxiety and depression than with symptoms of schizophrenia in one study [18]. Patients with PTSD and SP were also observed to have lower QoL and greater risk of lifetime suicide [4]. Across anxiety conditions, lower QoL was found in General Anxiety Disorder, SP and Panic Disorder [25, 30]. However, the Social Functioning domain was more impaired in SP compared to other anxiety conditions and amongst SP, QoL was negatively correlated with functional impairment and depressive features [25, 27].

## 4 DISCUSSION

There are several main findings from this review. First, the prevalence of SP varied from 2.1-7.1% amongst the community population, 12.7-17% amongst patients with psychiatric comorbidity and up to 37.6% amongst undergraduates. Second, correlates of SP included demographic (such as females, younger age, living alone, fewer years of education, unemployment) and clinical factors (such as family history of anxiety disorders, suicidal ideas, avoidant personality features). Third, SP was widely associated with decreased QoL involving several domains and especially related to complexity, greater number of feared or trigger situations, and comorbid medical and psychiatric conditions.

The prevalence of SP within community population (2.1%-7.1%) is consistent with findings of an earlier study which reported a rate of 5.8% [31]. Amongst individuals with co-occurring psychiatric conditions such as schizophrenia and PTSD, the rates of SP were higher (12.7%-17%), which behoove clinicians to be attentive to the possible presence of SP and address its management so as to optimise overall care for these patients. It is intriguing that the prevalence rates are even higher amongst undergraduates (up to 37.6%) from extant data, which is consistent with other studies which did not include examination of QOL amongst university students [32-34]. There could be several possible explanations. As SP is associated with younger age [13, 35], there is a naturally higher prevalence amongst the undergraduate population. Other correlates with SP included singlehood, living alone, and unemployment [6, 13, 26], which are common demographic features of these students including those that travel from far away to enroll for their university studies. Within the university, it may be the first time that the undergraduate student is exposed to a bigger group of colleagues, which can pose challenges to individuals with avoidant personality features [19]. In the course of study, their academic progress can also influence their condition as poor academic results [8] have been associated with SP. Of note, SP can be associated with a family history of anxiety disorders and suicidal ideas or attempts [8, 19]. This also highlights the need to identify SP and its associated risk factors amongst undergraduate learners early so that they can be encouraged to seek help whenever appropriate and needed. This can be a conjoint partnership between the students, faculty, university healthcare centres and involve psychoeducation about SP, de-stigmatisation, and providing access to psychiatric services and support of the learners.

Across mental disorders, the highest levels of disability and loss of QoL were found in people with three or more mental disorders in the last 12 months [36], with the loss of QoL showing a dose–response relationship with the number of mental disorders including SP [36]. Independently, SP was associated with lower QoL across the majority of studies and affected different domains including physical, psychological, and social aspects [3, 19]. It was one of the five mental disorders with the strongest association with poorer QOL [36], highlighting the extensive impact on the sense of well-being of the sufferers. The association of poor QoL with complex, generalised SP, and greater number of feared and trigger situations in SP underscore the direct relationship between severity of illness and overall sense of wellbeing. Of note, poor QOL had a stronger association with SP or anxiety-related factors as compared to depressive symptoms [26] or schizophrenia [18]. The association of SP with comorbid physical and psychiatric conditions reflects the incremental burden of medical and psychiatric illnesses, hence prompt and timely management of underlying and co-occurring conditions can ameliorate the overall morbidity, which can also improve the QoL [18]. This is evident from studies where patients who received treatment for their physical condition reported better QOL than those who had not [11, 20]. Sparse treatment studies with CBT, exposure therapy, and internet based self-help approaches suggested positive improvements of anxiety in SP as well as QoL over a period of follow-up [21-24]. This provides hope for individuals with SP that they can benefit from such psychotherapeutic and online approaches, which also improves QoL.

There are several limitations for this review. First, we limit the review to adults with SP and the impact on QoL, hence the data may not be generalisable to younger or much older individuals. Second, there are relatively fewer treatment studies. Third, there are also fewer prospective studies to evaluate the evolution of SP and inter-relationship with QoL and related factors over time.

Future studies may want to examine larger groups of SP and associated clinical correlations over time from different collaborating sites and compare wider settings to facilitate better generalisability of the findings. There is a need to investigate the effectiveness of current and future non-pharmacological treatments delivered over the virtual platform, which will facilitate the delivery of effective treatments for SP in spite of difficulties with face-to-face meetings due to various reasons such as distance, travel restrictions, and infection control measures.

## CONCLUSION

In conclusion, SP occurs not uncommonly within community, clinical and undergraduate populations and is often associated with poor QoL. The relationship between clinical features of SP and comorbidity with poor QoL warrants earlier identification of SP so that it can be addressed in a timely way with current and future treatment approaches.

## Figures and Tables

**Fig. (1):**
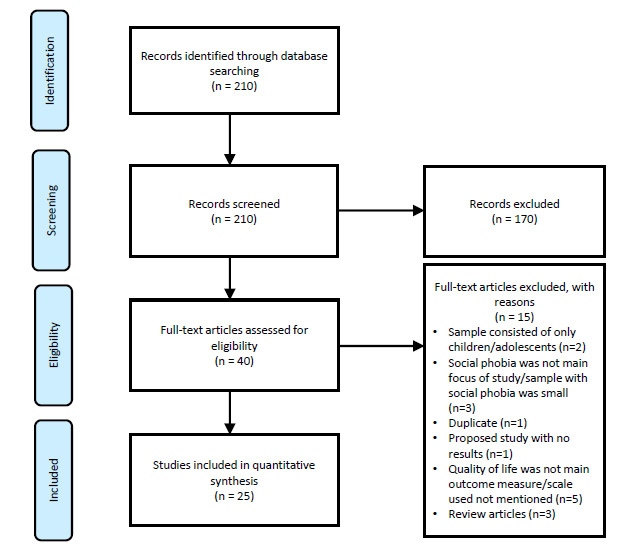
PRISMA flowchart of reviewed studies related to Quality of Life in adults with Social Phobia.

**Table 1:** Summary of relevant main findings from included studies assessing Quality of Life (QoL) in Social Phobia (SP).

Authors/Year/Country	Nature of Study/Demographic Features	Measure of QOL	Other Rating Tools used	Main Findings
Hajure & Abdu, 2020 Ethiopia [3]	Cross sectional study 523 undergraduate students Females 48.6% Mean age 22.07+/-2.36 years	WHOQOL-BREF	SP Inventory (SPIN); Liebowitz Social Anxiety Scale (LSAS)	25.8% had a SPIN score of 19 (cut off for SP) SP had significantly lower QoL quality than those without SP Being females, current tobacco use, and family history of psychiatric illness were factors significantly associated with SP
Kählke *et al.*, 2019 Europe [23]	Prospective study, follow up 6 months Two-arm randomized controlled design N = 200 university undergraduates with SP randomly allocated to an internet-based unguided CBT (n = 100) or to a 6-month WLC group (n = 100) Females 62% Mean age 26.70 +/-6.34 years	Assessment of QoL Scale	Beck Depression Inventory; Brief Symptom Inventory; Liebowitz Social Anxiety Scale; Inventory of Interpersonal Problems; German Client Satisfaction Questionnaire	Improvements in QoL after 6 months for intervention group
Kim *et al.*, 2019 Austria [11]	Prospective study, follow up 36 months 30 patients with Hemifacial Spasm Females 66.7% Mean age 51.6 ± 9.0 years	SF-36	Hospital Anxiety Depression Scale (HADS); Liebowitz Social Anxiety Scale (LSAS)	6 out of 22 patients who completed the study had SP At baseline, SP group showed greater impairment in the Role Physical, Vitality, Role Emotional, and Mental Health dimensions of the SF-36 Significant differences between baseline and 6 months and 36 months of follow-up assessments 6 months after MicroVascular Decompression (MVD), improvements of the SP group in HADS anxiety subscale, LSAS total score At 36 months, the improvement of the scales mentioned above was maintained, and additionally Vitality and Mental Health dimensions of SF-36 showed a statistically significant improvement
Canuto *et al.*, 2018 International study (Germany, Italy, England, Spain, Switzerland, and Israel) [35]	Cross-sectional study 3,142 participants in community Females 50.7% Mean age 73.7 years	WHOQOL-BREF		One in six older adults was diagnosed with anxiety disorders including SP The prevalence rate of any anxiety disorder dropped by 40% in adults 75-79 years old as compared to adults 65-69 years old. The prevalence rate dropped by 47% for those aged 75–84 years compared to those aged 65-69 years. Anxiety disorders often emerge at an early age and tend to wax and wane over the life cycle Weak association with QoL may show that people learn to live and cope with their anxieties
Joseph *et al.*, 2018 India [8]	Cross sectional study 450 undergraduate students Females 30.7% Mean age: 20.6±1.6 years	QoL Enjoyment and Satisfaction Questionnaire	SP Inventory (SPIN)	169 (37.6%) participants were found to have SP Family history of anxiety disorders, embarrassment with own socio-economic status and past history of failure in academic examinations were significantly associated with the presence of SP among the participants. Mean QoL scores correlated with increasing severity of SP
McMillan *et al.*, 2017 USA [4]	Cross sectional study 2443 participants in community PTSD no SP: n=1498 SP no PTSD: n=728 PTSD-SP: n=217 Females 66% Mean age: Not stated	SF-12	Physical Functioning - Physical Composite Score (PCS); Vitality, Social Functioning, Role limitations - Mental Composite Score (MCS)	Relative to those with either PTSD or SP, individuals with comorbid PTSD-SP demonstrated an elevated risk of lifetime suicide attempts and substantially lower levels of QoL.
Vrbova *et al.*, 2017 Czech Rep [5]	Prospective study 70 patients with schizophrenia and psychotic spectrum illnesses Females 51.7% Mean age 35.6 years Duration of psychotic illness 7.3 years	QoL Enjoyment and Satisfaction Questionnaire (Q-LES-Q)	Internalized Stigma of Mental Illness (ISMI) scale; Adult Dispositional Hope Scale (ADHS); Liebowitz Social Anxiety Scale (LSAS); Beck Anxiety Inventory (BAI); Beck Depression Inventory-II (BDI-II); Positive and Negative Syndrome Scale (PANSS); Temperament and Character Inventory – Revised (TCI-R)	Clinically, the patients with comorbid SP had poorer QOL, earlier onset of the illness, more severe current psychopathology, more intense anxiety (general and social), higher severity of depressive symptoms, lower level of hope, higher level of self-stigma, harm avoidance trait and lower score of self-directedness trait compared with patients without SP
Kampmann *et al.*, 2016 Netherlands [22]	Prospective study, follow up 4 months 60 participants SP subjects were randomly assigned to individual virtual reality exposure therapy (VRET), individual in vivo exposure therapy (iVET), or waiting-list. The average number of completed sessions was 8.50 (SD = 2.63) for VRET and 8.55 (SD = 2.68) for iVET. Females 63.3% Mean age 36.9 years	EuroHIS QoL Scale	Social Interaction Anxiety Scale; Liebowitz Social Anxiety Scale-Self Report (LSAS-SR); Fear of Negative Evaluation Scale-Brief Form (FNE-B); Depression Anxiety Stress Scale (DASS-21); Personality Disorder Belief Questionnaire (PDBQ)	EUROHIS-QOL showed significant increase from pre-to post assessment for iVET compared to the waiting-list control group over 4 months. No significant differences were found between VRET and the waiting-list control group.
Salman *et al.*, 2016 Switzerland [29]	Cross-sectional study 45 vitiligo and 48 acne patients participated from dermatology clinics Females 54.1% Mean Age: 27.05 years	Dermatology Life Quality Index (DLQI)	Liebowitz Social Anxiety Scale (LSAS); Hospital Anxiety and Depression Scale (HADS)	Social anxiety of vitiligo and acne patients were significantly higher than healthy controls QoL was negatively correlated with social anxiety in both patient groups.
Mirijello *et al.*, 2015 Italy [20]	Cross sectional study 60 morbidly obese patients (30 patients were waiting for surgical treatment in Grp A while 30 who had undergone bariatric surgery in Grp B) Females 69.6% Mean age 42.5+-10.9 50 Healthy Control group Females 80% Mean age 35.5+-10.5 years	SF-36	Liebowitz Social Anxiety Scale (LSAS); State and Trait Anxiety Inventory (STAI); Zung Self-rating Depression Scale (Zung-SDS); Body Shape Questionnaire (BSQ)	Percentage of patients showing SP was significantly higher compared to a sample of healthy controls (43% vs 16%; p=0.004), especially in those waiting for surgery versus those who had undergone surgery Patients awaiting surgery had lower QoL scores vs patients already treated by surgery. Males have lower propensity than females to show SP
Craske *et al.*, 2014 USA [21]	Prospective study, follow up 12 months 100 subjects with SP were randomized to ACT (n=34), CBT (n =40), or wait list (WL; n=26). Females 45.98% Mean age: 28.37 years	QoL Inventory	Liebowitz Social Anxiety Scale–Self-Report (LSAS–SR); Social Interaction Anxiety Scale (SIAS); SP Scale (SPS)	QoL significantly improved over the full 12-month period in treated participants
Wong *et al.*, 2012 USA [28]	Cross sectional study 379 adults - 200 NC, 119 Generalised SP (GSP), 60 Non-Generalised (NGSP) Females 53.8% Mean age 39.78 (NC)/36.24 years (GSP)/42.15 (NGSP), 39.05 years	QoL Questionnaire	Social Skills assessment; SP and Anxiety Inventory	GSP subtype reported worse QOL relative to NC/NGSP (equivalent QoL) Presence of comorbidity negatively affected QOL in individuals with NGSP SP and social effectiveness exert independent effects on QOL
Gültekin & Dereboy, 2011 Turkey [19]	Cross-sectional study 700 Undergraduate students Females 52.6% Mean age: 21.16 ±1.76 years	WHOQOL BREF	Liebowitz Social Anxiety Scale (LSAS)	Students with SP had significantly lower QoL than those without SP. Generalized SP had significantly higher LSAS anxiety, and avoidant behavior scores than those with a specific SP SP associated with females, living in cities for the last 15 years, those that had relatives with a psychiatric illness, suicidal ideation
Comer *et al.*, 2011 USA [12]	Cross-sectional study 43,093 respondents in community, out of which 1,140 with social anxiety disorder Females 63.3% (social anxiety disorder group) Mean age: Not stated	SF-12		The 12-month prevalence estimate of any of the four DSM-IV anxiety disorders was 9.8%, SP was 7.1% SP was correlated with poorer QoL (impaired social and role functioning, mental health, and overall physical and mental well-being)
Barrera & Norton, 2009 USA [25]	Cross-sectional study 67 subjects, out of which 27 subjects with SP Females 62.7% (overall) Mean age: 33.83 years	QoL Inventory		Degree of QoL impairment was similar across SP, Generalised Anxiety Disorder and Panic Disorder SP reported poorer QoL than a normative control group, QoL was negatively correlated with functional impairment and depression, Social Functioning domain was more impaired in SP
Acarturk *et al.*, 2008 Netherlands [13]	Cross-sectional study 7,076 persons in community 12-month prevalence of SP: 4.8% Females 63.2% (SP group), 48.7% (no SP group) Mean age: 40.2 (SP group), 41.2 (no SP group)	SF-36		The 12-month prevalence of SP was 4.8%, and significantly associated with being female, of a younger age, being less well educated, and living alone 66% of respondents with SP had at least one comorbid condition and associated lower QoL and higher service utilization. SP was especially strongly associated with obsessive compulsive disorder, bipolar disorder, agoraphobia without panic disorder, and panic disorder. Number of social fears was negatively associated with QoL, and was an important determinant of the severity of SP
Braga *et al.*, 2005 Brazil [18]	Cross-sectional study 53 patients with schizophrenia Females with comorbid anxiety disorder 31.8% Mean age: 35.5 years	Sheehan disability scale (SDS)	Brief Psychiatric Rating Scale (BPRS)	Lifetime prevalence of SP 17%, associated with poorer QoL Low QoL was more strongly associated with high rates of anxiety and depression than with any other symptoms of schizophrenia
Rapaport *et al.*, 2005 USA [37]	Cross-sectional study 67 persons in community Females 65.8% Mean age: 32.4 years 358 patients with social phobia Females 40% Mean age: 35.5 years	Baseline QoL Enjoyment and Satisfaction Questionnaire	Liebowitz Social Anxiety Scale (LSAS)	Anxiety disorders including SP had lower QoL vs community norm across all domains
Cramer *et al.*, 2005 USA [30]	Cross-sectional study 2065 subjects in community 283 with SP (lifetime) Females: Not stated Mean age: Not stated	QOL was assessed during interview based on 7 aspects: Subjective well-being, self-realisation, Contact with friends, Support if ill, absence of negative life events, relation to family of origin, neighbourhood quality, QoL		SP and panic disorder within the past year and lifetime, and generalised anxiety disorder within the past year, had an independent effect on QoL when controlling for a number of sociodemographic variables, somatic health and other DSM-III-R Axis I mental disorders. SP has the strongest impact on global QoL, especially self-realisation and contact with friends.
Alonso *et al.*, 2004 European countries [36]	Cross-sectional study 21 425 respondents in community Females 52% Mean age 47 years	SF-12	WHO Disablement Assessment Scale version 2 (WHODAS-II)	Five mental disorders with the strongest association with lower QoL and substantial level of disability: dysthymia, major depressive episode, PTSD, panic disorder and SP
Quilty *et al.*, 2003 Canada [27]	Cross-sectional study 360 subjects at seen at anxiety clinic Females 64.4% Mean age 33.9+/-10.4 years Social phobia 25%	Medical Outcome Study (MOS) Health Survey, self report covering 8 domains Final two-factor model used: Occupational Functioning and Relationships / Activities	Sheehan Disability Scale; Social Adjustment Scale-Self Report; Liebowitz Disability Self-Rating Scale; Beck Depression Inventory (BDI); State-Trait Anxiety Inventory (STAI)	SP correlated negatively with QoL-relevant subscales: role functioning due to emotional problems and social functioning Individuals with SP are more impaired in their Relationships/Activities than individuals with Panic Disorder.
Stein & Kean, 2000 Canada [14]	Cross-sectional study 8,116 subjects in community, out of which 1,116 with SP (lifetime) Females 60.6% (SP group) Mean age: Not stated	Quality of Well-Being Scale		One-year and lifetime prevalence rates of SP were 6.7% and 13.0% respectively, associated with extensive functional disability, less life satisfaction, and a lower quality of well-being, especially with complex fears SP subtype Lifetime SP was associated with being female, being young, lifetime major depression, failing a grade, not significantly associated with social class or personal income
Wittchen *et al.*, 1999 Germany [6]	Cross-sectional study 150 subjects with SP (65 had current pure SP and no comorbidity 51 comorbid subjects with current threshold SP - All had lifetime or current diagnoses of major depression and/or dysthymia. Most had additional lifetime diagnosis. 34 cases of subthreshold SP (All diagnostic criteria except current impairment) 65 Healthy Controls Females: 63.1% (pure SP), 60.8% (comorbid), 58.8% (subthreshold), 63.1% (controls) Mean age: 36.9 (pure SP), 38.0 (comorbid), 35.4 (subthreshold), 37.2 (controls)	SF-36	Liebowitz disability self-rating scale (LDSRS) Health service use inventory (HSUI)	QOL was significantly reduced in SP, particularly in scales measuring vitality, general health, mental health, role limitations due to emotional health, and social functioning
Safren *et al.*, 1996 USA [24]	Cross-sectional study 44 subjects at clinic Females 65% Mean age 35.80+/-10.42 years	QoL Inventory (QOLI)	Liebowitz Social Anxiety Scale (LSAS); Clinical Global Impression; Social Interaction Anxiety Scale (SIAS); SP Scale (SPS)	QoL of SP was significantly lower than the other patients, esp in singles/divorced/separated, improved with CBT group treatment for SP
Wittchen & Beloch, 1996 Germany [26]	Cross-sectional study 65 Pure SP, Females 63.1%, Mean age 36.9 years 65 Controls, Females 63.1%, Mean age 37.2 years	SF-36	Reilly Work Productivity and Impairment questionnaire (WPAI) Health Service Use Inventory (HSUI)	SP associated with lower QOL especially vitality, general health, mental health, role limitations due to emotional health and social functioning. WPAI was significantly diminished in SP Number of SP trigger situations was found to be strongest predictor of QoL reductions, not duration of illness, age of onset, depression

**Abbreviations:** QoL = Quality of life; SF-12 = Short Form-12; SF-36 = Short Form-36; WHOQOL-Bref = World Health Organisation QoL - Brief Form

## LIST OF ABBREVIATIONS

CBT: = Cognitive Behavioural Therapy
GSP: = Generalized Social Phobia
NGSP: = Non-Generalized Social Phobia
PTSD: = Post-Traumatic Stress Disorder
SF-36: = Short Form 36
SP: = Social Phobia
QoL: = Quality of Life

## References

[1] American Psychiatric Association (2013). Anxiety Disorders..

[2] Stein D.J., Lim C.C.W., Roest A.M., de Jonge P., Aguilar-Gaxiola S., Al-Hamzawi A., Alonso J., Benjet C., Bromet E.J., Bruffaerts R., de Girolamo G., Florescu S., Gureje O., Haro J.M., Harris M.G., He Y., Hinkov H., Horiguchi I., Hu C., Karam A., Karam E.G., Lee S., Lepine J.P., Navarro-Mateu F., Pennell B.E., Piazza M., Posada-Villa J., Ten Have M., Torres Y., Viana M.C., Wojtyniak B., Xavier M., Kessler R.C., Scott K.M., WHO World Mental Health Survey Collaborators (2017). The cross-national epidemiology of social anxiety disorder: Data from the World Mental Health Survey Initiative.. BMC Med..

[3] Hajure M., Abdu Z. (2020). Social phobia and its impact on quality of life among regular undergraduate students of Mettu University, Mettu, Ethiopia.. Adolesc. Health Med. Ther..

[4] McMillan K.A., Asmundson G.J.G., Sareen J. (2017). Comorbid PTSD and social anxiety disorder: Associations with quality of life and suicide attempts.. J. Nerv. Ment. Dis..

[5] Vrbova K., Prasko J., Ociskova M., Holubova M. (2017). Comorbidity of schizophrenia and social phobia - impact on quality of life, hope, and personality traits: A cross sectional study.. Neuropsychiatr. Dis. Treat..

[6] Wittchen H.U., Fuetsch M., Sonntag H., Müller N., Liebowitz M. (1999). Disability and quality of life in pure and comorbid social phobia--findings from a controlled study.. Eur. Psychiatry.

[7] Acarturk C., Smit F., de Graaf R., van Straten A., Ten Have M., Cuijpers P. (2009). Economic costs of social phobia: A population-based study.. J. Affect. Disord..

[8] Joseph N., Rasheeka V.P., Nayar V., Gupta P., Manjeswar M.P., Mohandas A. (2018). Assessment of determinants and quality of life of university students with social phobias in a coastal city of south India.. Asian J. Psychiatr..

[9] Bruce T.J., Saeed S.A. (1999). Social anxiety disorder: A common, underrecognized mental disorder.. Am. Fam. Physician.

[10] Heinig I., Wittchen H-U., Knappe S. (2021). Help-seeking behavior and treatment barriers in anxiety disorders: Results from a representative German community survey.. Community Ment. Health J..

[11] Kim Y.G., Chang W.S., Jung H.H., Chang J.W. (2019). The long-term effects of microvascular decompression on social phobia and health-related quality of life in patients with hemifacial spasm: A 3-year prospective study.. Acta Neurochir. (Wien).

[12] Comer J.S., Blanco C., Hasin D.S., Liu S.M., Grant B.F., Turner J.B., Olfson M. (2011). Health-related quality of life across the anxiety disorders: Results from the national epidemiologic survey on alcohol and related conditions (NESARC).. J. Clin. Psychiatry.

[13] Acarturk C., de Graaf R., van Straten A., Have M.T., Cuijpers P. (2008). Social phobia and number of social fears, and their association with comorbidity, health-related quality of life and help seeking: A population-based study.. Soc. Psychiatry Psychiatr. Epidemiol..

[14] Stein M.B., Kean Y.M. (2000). Disability and quality of life in social phobia: Epidemiologic findings.. Am. J. Psychiatry.

[15] Peters M.D.J., Godfrey C.M., Khalil H., McInerney P., Parker D., Soares C.B. (2015). Guidance for conducting systematic scoping reviews.. Int. J. Evid.-Based Healthc..

[16] Arksey H., O’Malley L. (2005). Scoping studies: Towards a methodological framework.. Int J Soc Res Methodol Theory Pract..

[17] Moher D., Liberati A., Tetzlaff J., Altman D.G., PRISMA Group (2009). Preferred reporting items for systematic reviews and meta-analyses: The PRISMA statement.. BMJ.

[18] Braga R.J., Mendlowicz M.V., Marrocos R.P., Figueira I.L. (2005). Anxiety disorders in outpatients with schizophrenia: Prevalence and impact on the subjective quality of life.. J. Psychiatr. Res..

[19] Gültekin B.K., Dereboy I.F. (2011). The prevalence of social phobia, and its impact on quality of life, academic achievement, and identity formation in university students.. Turk Psikiyatr. Derg..

[20] Mirijello A., D’Angelo C., Iaconelli A., Capristo E., Ferrulli A., Leccesi L., Cossari A., Landolfi R., Addolorato G. (2015). Social phobia and quality of life in morbidly obese patients before and after bariatric surgery.. J. Affect. Disord..

[21] Craske M.G., Niles A.N., Burklund L.J., Wolitzky-Taylor K.B., Vilardaga J.C.P., Arch J.J., Saxbe D.E., Lieberman M.D. (2014). Randomized controlled trial of cognitive behavioral therapy and acceptance and commitment therapy for social phobia: Outcomes and moderators.. J. Consult. Clin. Psychol..

[22] Kampmann I.L., Emmelkamp P.M.G., Hartanto D., Brinkman W.P., Zijlstra B.J.H., Morina N. (2016). Exposure to virtual social interactions in the treatment of social anxiety disorder: A randomized controlled trial.. Behav. Res. Ther..

[23] Kählke F., Berger T., Schulz A. (2019). Int J Methods Psychiat Res..

[24] Safren S.A., Heimberg R.G., Brown E.J., Holle C. (1996-1997). Quality of life in social phobia.. Depress. Anxiety.

[25] Barrera T.L., Norton P.J. (2009). Quality of life impairment in generalized anxiety disorder, social phobia, and panic disorder.. J. Anxiety Disord..

[26] Wittchen H.U., Beloch E. (1996). The impact of social phobia on quality of life.. Int Clin Psychopharmacol.

[27] Quilty L.C., Van Ameringen M., Mancini C., Oakman J., Farvolden P. (2003). Quality of life and the anxiety disorders.. J. Anxiety Disord..

[28] Wong N., Sarver D.E., Beidel D.C. (2012). Quality of life impairments among adults with social phobia: The impact of subtype.. J. Anxiety Disord..

[29] Salman A., Kurt E., Topcuoglu V., Demircay Z. (2016). Social anxiety and quality of life in vitiligo and acne patients with facial involvement: A cross-sectional controlled study.. Am. J. Clin. Dermatol..

[30] Cramer V., Torgersen S., Kringlen E. (2005). Quality of life and anxiety disorders: A population study.. J. Nerv. Ment. Dis..

[31] Reddy V.M., Chandrashekar C.R. (1998). Prevalence of mental and behavioural disorders in India : A meta-analysis.. Indian J. Psychiatry.

[32] Bella T.T., Omigbodun O.O. (2009). Social phobia in Nigerian university students: Prevalence, correlates and co-morbidity.. Soc. Psychiatry Psychiatr. Epidemiol..

[33] Baptista C.A., Loureiro S.R., de Lima Osório F., Zuardi A.W., Magalhães P.V., Kapczinski F., Filho A.S., Freitas-Ferrari M.C., Crippa J.A. (2012). Social phobia in Brazilian university students: Prevalence, under-recognition and academic impairment in women.. J. Affect. Disord..

[34] Tillfors M., Furmark T. (2007). Social phobia in Swedish university students: Prevalence, subgroups and avoidant behavior.. Soc. Psychiatry Psychiatr. Epidemiol..

[35] Canuto A., Weber K., Baertschi M., Andreas S., Volkert J., Dehoust M.C., Sehner S., Suling A., Wegscheider K., Ausín B., Crawford M.J., Da Ronch C., Grassi L., Hershkovitz Y., Muñoz M., Quirk A., Rotenstein O., Santos-Olmo A.B., Shalev A., Strehle J., Wittchen H.U., Schulz H., Härter M. (2018). Anxiety disorders in old age: Psychiatric comorbidities, quality of life, and prevalence according to age, gender, and country.. Am. J. Geriatr. Psychiatry.

[36] Alonso J., Angermeyer M.C., Bernert S., Bruffaerts R., Brugha T.S., Bryson H., de Girolamo G., Graaf R., Demyttenaere K., Gasquet I., Haro J.M., Katz S.J., Kessler R.C., Kovess V., Lépine J.P., Ormel J., Polidori G., Russo L.J., Vilagut G., Almansa J., Arbabzadeh-Bouchez S., Autonell J., Bernal M., Buist-Bouwman M.A., Codony M., Domingo-Salvany A., Ferrer M., Joo S.S., Martínez-Alonso M., Matschinger H., Mazzi F., Morgan Z., Morosini P., Palacín C., Romera B., Taub N., Vollebergh W.A., ESEMeD/MHEDEA 2000 Investigators, European Study of the Epidemiology of Mental Disorders (ESEMeD) Project (2004). Disability and quality of life impact of mental disorders in Europe: Results from the European Study of the Epidemiology of Mental Disorders (ESEMeD) project.. Acta Psychiatr. Scand. Suppl..

[37] Rapaport M.H., Clary C., Fayyad R., Endicott J. (2005). Quality-of-life impairment in depressive and anxiety disorders.. Am. J. Psychiatry.

